# Assessing Diagnostic Accuracy of Haemoglobin Colour Scale in Real-life Setting

**Published:** 2014-03

**Authors:** Pankaj P. Shah, Shrey A. Desai, Dhiren K. Modi, Shobha P. Shah

**Affiliations:** SEWA-Rural, Jhagadia, Bharuch district, Gujarat 393 110, India

**Keywords:** Anaemia, Community health worker, Haemoglobin Colour Scale, Sensitivity, Specificity

## Abstract

The study was undertaken to determine diagnostic accuracy of Haemoglobin Colour Scale (HCS) in hands of village-based community health workers (CHWs) in real-life community setting in India. Participants (501 women) were randomly selected from 8 villages belonging to a project area of SEWA-Rural, a voluntary organization located in India. After receiving a brief training, CHWs and a research assistant obtained haemoglobin readings using HCS and HemoCue^TM^ (reference) respectively. Sensitivity, specificity, positive and negative predictive-values, and likelihood ratios were calculated. Bland-Altman plot was constructed. Mean haemoglobin value, using HCS and HemoCue^TM^ were 11.02 g/dL (CI 10.9-11.2) and 11.07 g/dL (CI 10.9-11.2) respectively. Mean difference between haemoglobin readings was 0.95 g/dL. Sensitivity of HCS was 0.74 (CI 0.65-0.81) and 0.84 (CI 0.8-0.87) whereas specificity was 0.84 (CI:0.51-0.98) and 0.99 (CI:0.97-0.99) using haemoglobin cutoff limits of 10 g/dL and 7 g/dL respectively. CHWs can accurately diagnose severe and moderately-severe anaemia by using HCS in real-life field condition after a brief training.

## INTRODUCTION

Iron deficiency is the most common micronutrient deficiency in the world, affecting 2 billion people (1). Anaemia causes 841,000 deaths and loss of 35,057,000 disability-adjusted life years (DALYs) globally every year (2). The first logical step to control anaemia should be to have an accurate diagnosis of anaemia at community and clinical settings (3). Clinical examination for pallor is the most commonly-used method for anaemia detection. Some of the other commonly-used methods to estimate haemoglobin in a community setting are: Sahli method, copper sulphate method, and HemoCue^TM^. Unfortunately, these methods have several limitations, ranging from lack of accuracy to complexity to high cost (4).

To overcome many of these problems, Haemoglobin Colour Scale (HCS) was introduced by the World Health Organization to be used in community setting for estimation of haemoglobin. HCS does not require battery or electricity or any maintenance. HCS is portable, and the results are immediate (5). The cost for performing one test is approximately 10 cents.

Accuracy of Haemoglobin Colour Scale is well-established when used in ideal laboratory conditions by a trained technician. Its accuracy is largely unknown in real-life community settings when used by village-based community health workers.

There have been a number of studies, examining the accuracy of HCS in various parts of the world (6-15). Many of these studies are done in ideal conditions in laboratories by trained laboratory technicians. Selected community-based studies revealed a large variation in accuracy of the HCS. Sensitivity and specificity of the HCS from five laboratory-based studies were all high, varying from 0.85 to 0.99 for sensitivity and from 0.91 to 1.0 for specificity. Sensitivity for the ‘real-life’ studies varied between 0.50 and 0.88 (16). Real-life setting means performing haemoglobin measurement under field condition by village-based community health workers (CHWs).

There is need for a study that would establish accuracy of HCS in hands of CHWs at the community level. Objective of this study is to determine diagnostic accuracy of HCS in hands of village-based CHWs in a community setting.

The indicators of diagnostic accuracy of HCS that were examined included the following:

Sensitivity, specificity, positive predictive value (PPV), negative predictive value (NPV), likelihood ratios (positive and negative), and efficiency of HCS compared to reference standard for diagnosis of anaemia and according to severity of anaemia; mean difference between readings obtained from HCS and reference standard and construct Bland-Altman plot with 95% limits of agreement; prevalence of anaemia and various severity of anaemia according to HCS and reference standard; mean difference between readings obtained from HCS and the reference method and construct Bland-Altman plot with 95% limits of agreement; and prevalence of anaemia and various levels of severity of anaemia according to HCS and the reference method.

## MATERIALS AND METHODS

### Study participants

This study was conducted among 8 randomly-selected villages of Jhagadia block located in Gujarat, India. Jhagadia block consists of 168 villages with a population of 171,000. Almost 70% of population is tribal. Most of the population is poor and involved in farming. This study was done by a local, voluntary organization known as SEWA-Rural (SR) which has provided community-based health services in this area for the last 30 years. SR provides community-based safe motherhood and newborn survival services among all villages of the Jhagadia block since 2003 through 168 trained CHWs. These CHWs are village-based workers who have received at least primary education and have good rapport and strong relationships within the community as they are native of the village. The CHWs are part of the primary healthcare system and had received short training to implement community-level interventions for improving maternal and child health.

One village was randomly selected from the 8 Primary Health Centres affiliated to cluster of villages belonging to Jhagadia block. Women of reproductive age-group between 15 and 45 years were included in this study. The sample included pregnant and lactating women. Participants were recruited randomly, using a household survey, based on the database of SEWA-Rural. Alternative participants were randomly chosen if previously-selected participants refused to participate. Required sample-size was 489 to predict sensitivity and specificity of the severity of anaemia at 0.1 precision with 95% confidence level. Data were collected prospectively.

### Test methods

Haemoglobin measurement using HemoCue^TM^ portable haemoglobinometer (HemoCue^TM^, ANGELHOLM, Sweden) was used as reference method. It has been demonstrated that HemoCue^TM^ can provide accurate haemoglobin estimation when compared with filter photometer (17,18). HemoCue^TM^ has been used for haemoglobin estimation during the Demographic and Health Survey (19). Considering accuracy and ease of use in field conditions, we chose HemoCue^TM^ as the reference method for our study. Supervisors of SR underwent training on the use of HemoCue^TM^.

The CHWs in the selected villages underwent a brief half-day training on proper use of the HCS. The HCSs were procured from the WHO-validated source *COPACK GmbH,* Germany. CHWs had hands-on training in addition to lectures. A training video prepared by SR was shown to the CHWs. In addition, a training module with pictorials was also provided.

Independent observer received training to ensure that protocols are implemented; CHWs and their supervisors were blind to result of the test done by the other individuals. Observers provided treatment and counselling to women who were found to be anaemic.

The CHWs and their supervisors measured haemoglobin of the same woman, using HCS and HemoCue^TM^ respectively after obtaining informed consent. The CHWs and supervisors were not in the same room to ensure that CHW's result does not get influenced by Hemocue^TM^ reading. First, the supervisor obtained capillary blood by pricking left ring-finger and measured haemoglobin, using HemoCue^TM^. After a few minutes, CHW obtained capillary blood by pricking right ring-finger and measured haemoglobin by using HCS. Both CHW and supervisor noted down the results in separate data-collection form. An independent observer ensured that CHW and supervisor do not disclose their findings to each other. All sharp objects were collected and disposed of according to accepted standards. The observer informed haemoglobin reading obtained from HemoCue^TM^ to the participant and provided further treatment and counselling according to predefined protocol. Severely-anaemic participants were referred to the hospital of SEWA-Rural. The observer brought the data-collection forms to SEWA-Rural where information was entered into Microsoft Excel sheet by one of the investigators.

We defined various levels of severity of anaemia as indicated in Table 1 for our study (20).

**Table 1.** Definition of severity of anaemia

Haemoglobin level (g/dL)	Severity
More than 12	Normal
12-10.1	Mild
10-7.1	Moderate
Less than or equal to 7	Severe

The study was approved by the Anusandhan Trust Ethical Review Committee, Mumbai.

### Statistical analysis and reporting

Statistical analysis was done using STATA 10.0 (21). Prevalence of anaemia according to various levels of severity was calculated. Paired *t*-test was performed to determine significant difference between means of haemoglobin values obtained by both the methods. Pearson's correlation coefficient was calculated. Mean difference in haemoglobin values obtained using HCS and HemoCue^TM^ with 95% confidence interval was recorded. Sensitivity, specificity, PPV, NPV, and efficiency with 95% confidence interval for detecting various severity levels of anaemia were calculated. Area under Receiver Operating Curve (ROC) was calculated using STATA command *lroc*. Likelihood ratios (positive and negative) were calculated. Bland-Altman plot was constructed using STATA command *batplot, notrend*. STARD guidelines were used for describing methods, statistical analysis, and results of the study (22).

## RESULTS

A total of 501 participants were recruited from 8 villages from March to May 2011. The description of study participants is provided in Table 2. Haemoglobin readings from HemoCue^TM^ and HCS were available for all 501 participants. Mean age of CHWs was 31 years. Five CHWs had education less than or equal to 10th grade. Mean duration of experience as CHW was 6.5 years.

**Table 2.** Description of study participants (n=501)

Characteristics	Values
Age (years)	
Mean	31
Median	30
Range	15 to 45
Caste	
Scheduled tribe	282 (56%)
Others	219 (44%)
Pregnancy status	
Pregnant	25 (5%)
Lactating	23 (5%)
Others	453 (90%)

Table 3 shows prevalence of anaemia in addition to summary statistics for haemoglobin readings using HemoCue^TM^ and HCS. There was no statistical difference between mean haemoglobin readings obtained from the two methods. Prevalence of anaemia was significantly higher (91%) when HCS was used compared to HemoCue^TM^ (71%), although this difference gradually reduced with increasing severity of anaemia.

**Figure 1.**
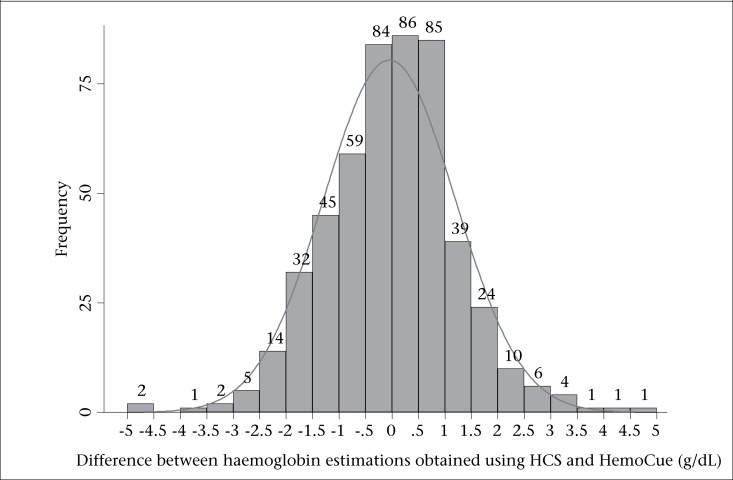
Difference between haemoglobin readings using HCS and HemoCue™

The mean difference between haemoglobin readings using HemoCue^TM^ and HCS was 0.95, after taking into account the exact value of the difference ignoring direction of difference from zero. Almost two-thirds (324/501) and 91% (454/501) haemoglobin readings obtained using HCS were within 1 g and 2 g of corresponding HemoCue^TM^ readings respectively; 11 (2%) readings obtained using HCS were beyond 3 g of HemoCue^TM^ readings. As seen in Figure 1, differences in haemoglobin readings were normally distributed around zero.

Table 4 shows sensitivity, specificity, PPV, NPV, likelihood ratio (positive and negative), and efficiency of HCS at different cutoff levels. Sensitivity and specificity of HCS for diagnosis of anaemia was 0.96 (CI 0.93-0.98) and 0.22 (CI 0.15-0.3) respectively. Almost all indicators of test accuracy increased with increasing severity of anaemia. Sensitivity of HCS was 0.74 (CI 0.65-0.81) and 0.84 (CI 0.8-0.87) whereas specificity was 0.84 (CI 0.51-0.98) and 0.99 (CI 0.97-0.99), using haemoglobin cutoff limits of 10 g/dL and 7 g/dL respectively. Log-likelihood (positive) ratio for severe anaemia was 83; therefore, correct diagnosis of severe anaemia was 83 times common if HCS test result was less than 7 g/dL. Log-likelihood (positive) ratio for haemoglobin less than 10 g/dL was 4.63; therefore, correct diagnosis of moderately-severe anaemia was 4.6 times common if HCS test result was less than 10 g/dL. In other words, diagnostic accuracy of HCS was more for severe anaemia than moderately-severe anaemia, and so on.

**Figure 2.**
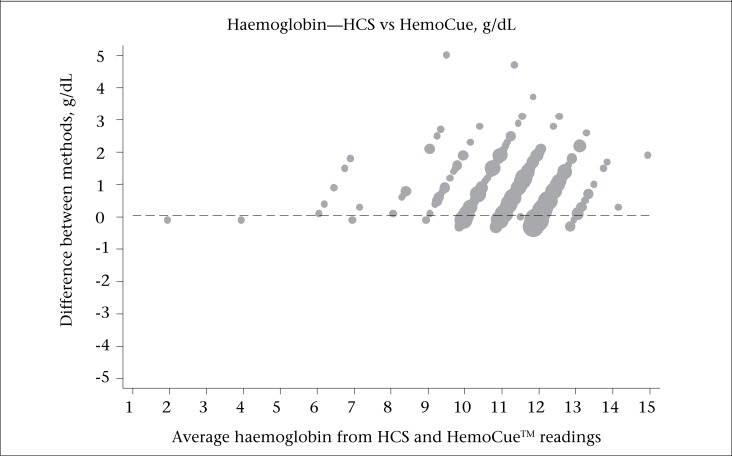
Bland-Altman plot of the haemoglobin estimates obtained using HCS and HemoCue^™^ (reference)

Bland-Altman plot (Figure 2) shows that mean difference between haemoglobin estimations obtained using HCS and HemoCue^TM^ was 0.05 g/dL with 95% limits of agreement from −2.38 to 2.49 g/dL. There was no relationship between difference in haemoglobin estimates obtained using both the methods and mean values. Pearson's correlation coefficient comparing HemoCue^TM^ and HCS was 0.7, 0.75, and 0.87 for haemoglobin cutoff level of less than or equal to 12, 10, and 7 respectively. Area under ROC was 0.79 for anaemia cutoff limit 10 g/dL, which suggests that there is a good agreement between HemoCue^TM^ and HCS.

**Table 3.** Prevalence of anaemia (n=501)

Severity of anaemia	HemoCue^TM^	HCS
Haemoglobin value (g/dL)
Mean	11.07 (CI 10.9-11.2)	11.02 (CI 10.9-11.2)
Median	11.03	11.00
Range	1.9-15.9	2-14
Anaemia (≤12 g/dL)	358 (71%)	456 (91%)
Mild anaemia (10.1-12 g/dL)	250 (50%)	312 (62%)
Moderately-severe anaemia (7.1-10 g/dL)	96 (19%)	130 (26%)
Severe anaemia (7 g/dL or less)	12 (2%)	14 (3%)

**Table 4.** Difference between readings using HemoCue^TM^ and HCS, and accuracy of HCS at different cutoff levels of haemoglobin (n=501

Parameter	Less than or equal to 12 g/dL	Less than or equal to 10 g/dL	Less than or equal to 7 g/dL
Sensitivity	0.96 (CI 0.93-0.98)	0.74 (CI 0.65-0.81)	0.83 (CI 0.51-0.98)
Specificity	0.22 (CI 0.15-0.3)	0.84 (CI 0.8-0.87)	0.99 (CI 0.97-0.98)
PPV	0.75 (CI 0.71-0.79)	0.56 (CI 0.47-0.64)	0.71 (CI 0.43-0.9)
NPV	0.69 (CI 0.53-0.81)	0.92 (CI 0.89-0.95)	0.99 (CI 0.98-1.0)
Likelihood ratio (positive)	1.23	4.63	83
Likelihood ratio (negative)	0.18	0.31	0.17
Efficiency	75%	82%	99%
Correlation coefficient	0.7	0.75	0.87

None of the participants suffered any harm due to their participation in the study.

## DISCUSSION

Clinical examination is the most commonly-used methods for diagnosis of anaemia at the community level. However, there is a fair volume of literature, suggesting inadequate accuracy of clinical examinations for detection of anaemia, especially non-severe anaemia (16,23-26). HCS was introduced by WHO to tackle this problem. Although HCS was found to be accurate in laboratory setting, very few studies examined its accuracy in hands of CHWs in a community setting where no laboratory facilities are available (16). This study tries to fill this gap in this evidence. This is the first study in India which examines accuracy of HCS in hands of village-based CHWs in a community setting.

For cutoff level at 12 g/dL, HCS was found to have high sensitivity (96%) but poor specificity. This means that HCS would misdiagnose many non-anaemic patients as anaemic and subject them to treatment. However, the treatment for mild anaemia consists of daily iron supplementation which is already a recommended preventive modality for non-anaemic pregnant women in India and is less likely to place any significant risk. Although there was no statistical difference in mean haemoglobin readings overall, correlation (0.7) between HemoCue^TM^ and HCS was fair. Most (91%) of HCS readings were within 2 g/dL of respective HemoCue^TM^ readings. This finding is in agreement with previous studies (16).

At cutoff level of 10 g/dL, accuracy of HCS was much better. Sensitivity (74%), specificity (84%), PPV (56%), NPV (92%), and correlation coefficient (0.75) was at acceptable range for community setting. Also, mean difference between HemoCue^TM^ and HCS improved (0.7 g/dL), and 98% of HCS readings were within 2 g/dL range of HemoCue^TM^. Area under ROC was 0.79. Also, HCS was accurately differentiating severely-anaemic participants from those suffering from moderate anaemia. This differentiation is important as management of severely-anaemic individual is different from moderately-anaemic individuals.

Sensitivity of HCS for diagnosis of anaemia varied from 50% to 86% in previous studies done under real-life condition (16). Three out of four real-life studies reported specificity of less than or equal to 50%. This study had similar findings, although sensitivity (96%) was better and specificity (22%) was worse than other real-life studies. This difference could be due to cutoff limits used for this study. Sensitivity analysis using haemoglobin level 11 g/dL as cutoff yielded sensitivity of 80% and specificity of 62%. More importantly, this study had much better sensitivity for severe anaemia than another real-life study which reported sensitivity of 50% (15).

It is important to compare accuracy of HCS with other field-based methods used for diagnosis of anaemia. Overall, clinical examination of pallor was found to have lower sensitivity but better specificity than HCS (6,13,15,27). Sahli and copper sulphate method had similar sensitivity and specificity as HCS (8,9,28). HemoCue^TM^ was used as the reference method for assessing accuracy of HCS in few studies. Sahli and copper sulphate methods are more complex than HCS as these require reagents and other preparations, which are tedious to perform in field setting. HemoCue^TM^ is the most expensive method as each test would cost almost 6 times compared to HCS. As with all invasive methods, the use of HCS carries risks associated with the use of lancets in a community setting if universal precautions are not implemented.

This study examined the validity of HCS. We cannot comment on the reliability of the method. There is a risk of CHW getting influenced by HemoCue^TM^ readings. We ensured that there were adequate physical barrier between CHWs and supervisor operating HemoCue^TM^. An independent observer was present at the time of data collection from every participant to ensure that CHW and supervisor were blinded to each other's results.

We recommend that HCS can be useful among communities which do not have easy access to laboratory facilities. To scale up the use of HCS, its use could be made part of syllabus of CHW training, and HCS could be made part of existing job-kit of CHWs. It would be important to overcome existing challenges related to replenishment of supplies, which is required for using HCS. Such policy would be a right step to achieve the Millennium Development Goal 5 (MDG 5) by early detection of anaemia which is one of the important indirect causes of maternal mortality and morbidity (29). More research is required to examine the utility of HCS for assessing response to the treatment for anaemia. Also, whether improved diagnosis of anaemia, using HCS, ultimately improves treatment and outcomes of anaemic patients remains to be examined.

What this study contributes to the existing literature is that village-level CHWs can accurately diagnose severe and moderately-severe anaemia, using HCS in community setting after a brief training. Accuracy of HCS in hands of CHWs is similar to what has been reported in previous studies where higher level of health workers, including laboratory technicians, midwives, used HCS. However, HCS does not replace laboratory assessment of anaemia.

### Conclusions

Haemoglobin Colour Scale is accurate in diagnosing severe and moderately-severe anaemia in high-prevalence area in hands of community health workers in real-life field condition, after a brief training.

## ACKNOWLEDGEMENTS

We are grateful to the John D and Catherine T MacArthur Foundation for their financial support. We would like to thank Dr. Rahul Shidhaye for his valuable suggestions for performing statistical analysis.
